# Root Anatomy and Root Canal Configuration of Human Permanent Mandibular Premolars: A Systematic Review

**DOI:** 10.1155/2013/254250

**Published:** 2013-12-22

**Authors:** Jojo Kottoor, Denzil Albuquerque, Natanasabapathy Velmurugan, Jacob Kuruvilla

**Affiliations:** ^1^Department of Conservative Dentistry and Endodontics, Mar Baselios Dental College, Kothamangalam, Ernakulam, Kerala 686691, India; ^2^Endodontic Speciality Practice, Dental Expert, Orlem, Malad, Mumbai 400064, India; ^3^Department of Conservative Dentistry and Endodontics, Meenakshi Ammal Dental College and Hospital, Alapakkam Main Road, Maduravoyal, Chennai 600 095, Tamil Nadu, India; ^4^Department of Public Health Dentistry, Mar Baselios Dental College, Kothamangalam, Ernakulam, Kerala 686691, India

## Abstract

*Introduction*. Mandibular premolars have been reported with complex anatomical aberrations, making them one of the most difficult teeth to manage endodontically. *Methodology*. An exhaustive search was undertaken to identify associated anatomic studies of mandibular premolars through MEDLINE/PubMed database using keywords, and a systematic review of the relevant articles was performed. Chi-square test with Yates correction was performed to assess the statistical significance of any anatomic variations between ethnicities and within populations of the same ethnicity. Documented case reports of variations in mandibular premolar anatomy were also identified and reviewed. *Results*. Thirty-six anatomic studies were analyzed which included 12,752 first premolars and nineteen studies assessing 6646 second premolars. A significant variation in the number of roots, root canals, and apical foramen was observed between Caucasian, Indian, Mongoloid, and Middle Eastern ethnicities.The most common anatomic variation was C-shaped canals in mandibular first premolars with highest incidence in Mongoloid populations (upto 24%) while dens invaginatus was the most common developmental anomaly. *Conclusions*. A systematic review of mandibular premolars based on ethnicity and geographic clusters offered enhanced analysis of the prevalence of number of roots and canals, their canal configuration, and other related anatomy.

## 1. Introduction

A clear understanding of the anatomy of human teeth is an essential prerequisite to all dental procedures especially so in the case of root canal treatment which deals with management of the tooth's internal anatomy. The pulp space is divided into two parts: the pulp chamber, which is usually described as that portion within the crown, and the pulp canal or root canal, which lies within the confines of the root. The pulp chamber is a single cavity; the dimensions of which vary according to the outline of the crown and the structure of the roots. In multirooted teeth the depth of the pulp chamber depends upon the position of the root furcation and may extend beyond the anatomical crown [1].

The pulp space is complex; root canals may divide and rejoin, and possess forms that are considerably more involved than commonly implied. Many roots have additional canals and a variety of canal configurations. In the simplest form, each root has a single canal and a single apical foramen (Type I). Commonly, however, other canal complexities are present and exit the root as one, two, or more apical canals (Types II–VIII) [2]. This could be better understood through an insight into the development of root formation. At a more advanced stage of tooth development, when enamel and dentin formation has reached the future cementoenamel junction, the dental root begins to form from a cellular diaphragm or horizontal Hertwig's epithelial root sheath. The horizontal Hertwig's epithelial root sheath may vary in shape, depending on whether the teeth are single- or multirooted. In fact, its shape determines the number of roots in a tooth. If the diaphragm remains in the shape of a collar, a single-rooted tooth will form. On the other hand, if two or three tongues of epithelium grow towards each other from this collar to bridge the gap and fuse, two or three diaphragms evolving independently from each other will form. They will either remain fused, forming fused roots, or single roots with multiple canals, or separated, forming distinct roots in multirooted teeth [3].

Mandibular premolars typically present with a single root and a single canal. The solitary root is usually oval in cross section containing an oval cross-section canal. Canal configurations in mandibular premolars may vary significantly with respect to ethnicity, race, and sex [2]. The purpose of this paper was to perform a systematic review of the literature related to the root anatomy and root canal configuration of the permanent mandibular first and second premolars.

## 2. Materials and Methods

### 2.1. Literature Search and Data Extraction

An exhaustive search was undertaken through MEDLINE/PubMed database to identify published literature related to the root anatomy and root canal morphology of the permanent mandibular premolars by using key words “root canal anatomy,” “root canal morphology,” “mandibular premolars,” “mandibular first premolar,” and “mandibular second premolar,” alone or in combination. Related anatomic studies of human permanent mandibular premolars were identified and a literature review was performed for articles dated July 2013 and before. The data was analyzed according to the population ethnicity and demography, number of teeth per study (power), number of roots, number of root canals, method of tooth analysis, root canal patterns, and number of apical foramina. Additionally, documented case reports of anatomic variations and developmental anomalies were identified and reviewed. Statistical comparisons were done between ethnicities and within populations of particular ethnicities using Chi-square test with Yates correction to allow for better understanding of the variations and the statistical significance of these variations in radicular anatomy of mandibular premolars based on the data collected through the systemic review.

## 3. Results

Thirty-six anatomic studies were analyzed which included 12,752 first premolars and nineteen studies of 6646 second premolars. A summary of the findings of different anatomic studies based on their ethnicity or geographical population assessment, with regard to the number of roots and canals has been tabulated for mandibular first premolars (Table 1) [4–38] and second premolars (Table 2) [4–7, 9, 11, 12, 15, 19, 21, 25, 29, 31–33, 35–40].

### 3.1. Radicular Anatomy with Ethnic and Demographic Patterns

In most instances, mandibular first premolars were found to have one root (97.21%). However, only 73.55% of these single rooted teeth contained a single canal. Thus, although the incidence of two roots was low (2.63%), about 23.55% teeth had two canals. Higher incidences of two roots were noted in the African-American population (16.2%) [14] and a Kuwaiti population (15%) [32]. Three or more rooted forms were reported in only 3% French [4] and 0.2% Indian [17] populations, respectively.

A higher incidence of two canals in mandibular first premolars was reported in several populations, upto 50% in Indian populations [16, 18–21] and approximately 40% in Middle Eastern populations from Kuwait [32], Jordan [33], and Turkey [36, 37]. While the Hispanic population [38] in Mexico also showed an incidence of 30.7%, the Chinese [22–27] and Caucasian [4, 6–14] populations had a variable incidence of two canals in about 10.7–36%. The incidence of three or more canals in mandibular first premolars was considerably lower (0–5%) [7, 12, 15, 22, 25, 27, 30, 33, 35, 36], with no such canal systems noted in Indian and Hispanic populations.

On the other hand, mandibular second bicuspids presented with a higher incidence of one root (99.28%) and one canal (86.9%). Overall, a second root was present in about 0.0–4.4% (average 0.61%) of teeth. In different studies, Caucasians [4, 6, 7, 9, 11, 12] and Turkish [36, 37] populations presented with a varied incidence of a second canal ranging from 2.5 to 34.4% and 6.4 to 29%, respectively. Second premolars presented with a second canal in Indian (13.5–20%) [19, 21], Iranian (5.8–17.5%) [35, 39, 40], or Jordanian (22.8%) [33] populations. However, Mongoloid [25, 29] and Hispanic [38] populations presented a much lower incidence of a second canal, 2% and 1.2%, respectively. Three or more canals in mandibular second premolars were scarcely reported (0–2%) [12, 15, 33].

C-shaped canal anatomy has been mainly documented in mandibular first bicuspids. Studies have reported a high incidence of C-shaped canals in Chinese populations ranging from 18 to 24% [23, 28], while other Chinese studies [25–27] have reported a lower incidence of 0–4–1.1%. In an Indian population, V. K. Sikri and P. Sikri [21] reported 10% first premolars exhibiting C-shaped canals while Sandhya et al. [16] reported the variation in 2% teeth in a southern Indian population. C-shaped canals were found to be of low incidence in second bicuspids, with only 0.6% and 2% incidence reported, respectively, in a Chinese [25] and an Iranian [35] study.

Lu et al. [23] coined the term “circumferential canals,” for an unusual aberration found in 6% of mandibular first premolars. A circumferential canal was described as a single canal in the center with 3 or 4 canals at the circumference when viewed in cross-section, that is, a single canal splitting into several canals (apical delta) at apical 3 mm from sagital view. The teeth had a single oval canal or two canals in an oval-shaped root while the C-shaped morphology was found in the apical 3 mm and/or 6 mm level cross-sections.

In both premolars, single canals were most likely to be an independent canal from orifice to apical foramen (Type I). However, in first premolar, a significant number of bifid canals fused prior to exit (Type II) in Indian and Turkish populations (9–18%) [16–21, 37]. Type IV pattern was more prevalent than Type V pattern in most population groups. However, Type V pattern (9–22%) was much higher than Type IV patterns (0–10%) in Mongoloids pointing to a higher prevalence of a singular canal bifurcating along its length, somewhat similar to the description of the above-mentioned circumferential canals. In second premolars, the Type V pattern was more prevalent than Type IV in all population groups, with significantly higher prevalence of upto 15–17% in Indian and Jordanian. A single apical foramen was present in 81% first premolars and 90% second premolars. The mandibular first premolar was more prone to bifurcation of canals (23–30%) terminating in multiple apical foramina (15–20%).

Based on the statistical analysis performed, a significant variation in the number of roots, root canals, and apical foramen was observed between all the ethnicities, in both mandibular bicuspids. Thus, based on this analysis, the number of roots, root canals, and apical foramen were significantly different in Caucasian, Indian, Mongoloid, and Middle Eastern populations. Additionally, there was also a significant difference in the root canal configurations of these population groups. However, in case of comparing for anatomic variations within populations of the same ethnicity, statistics could be performed only when sufficient data allowed for statistical analysis. The numbers of roots and root canals in first and second premolars were statistically significantly different in French, German, Polish, and the United States populations of Caucasians, as well as among the first premolars of Middle Eastern populations, Turkey, Jordan, Kuwait, and Iran. However, no such statistical significance was observed between Chinese and Korean second premolars.

Six *ex vivo* studies have reported the presence of a deep external mesial invagination (mesial invagination) along the root surface of mandibular premolars [16, 18, 20, 24, 33, 42] (Table 3). Sandhya et al. [16] assessed the root dentin thickness at the depth of the mesial invagination in mandibular first premolars and reported the average root thickness at the cervical, middle, and apical thirds to be 0.8, 0.78, and 0.3 mm, respectively. All C-shaped configuration premolars could also present an associated groove or concavity on the proximal lingual area of the middle root that would not always extend to the root apex. Some grooves presented as deep, folding grooves while others were not so distinguished or were just shallow concavities [23].

### 3.2. Gender Predilection

There is little documentation correlating the influence of gender on root/canal anatomy and its variations. Of the reported studies, females had a higher likelihood of two or more roots or canals in mandibular first premolars, whereas men exhibited multiple canals much more frequently than females in mandibular second premolars [17, 37, 43]. Others have reported no significant difference in root configuration between females and males [29].

### 3.3. Case Reports

In addition to these numerous population studies, thirty-six case reports have also documented anatomic variations. The anatomic aberrations reported as endodontic case reports in the literature include mandibular first premolars with 2 or 3 canals in 1 root or 2 roots; 3 roots and 3 canals; and 4 canals in 4 roots (Table 4) [44–59]. Mandibular second premolars have shown variations that include 2, 3, 4, and 5 canals in 1 root; 2, 3, and 4 canals in 2 roots; 3 roots and 3 or 4 canals; (Table 5) [46, 49, 53–55, 60–80].

### 3.4. Anatomic Developmental Anomalies

Adding to the complexity of the mandibular premolars are various developmental anomalies (Table 6) [77, 81–90]. In 1997, Hartup [81] reported a Type III dens invaginatus and a bifurcated root of the mandibular first premolar. Tavano et al. [83] reported a dens invaginatus wherein the clinical crown was larger than the contralateral first premolar. No case reports of dens invaginatus in the mandibular second premolar have been reported (Table 6). Conversely, dens evaginatus most frequently affected mandibular second premolars and was more often reported in Mongoloid people [88].

Aryanpour et al. [84] reported root canal and periodontal treatment of a geminated mandibular first premolar, that is, two distinct crowns with united roots, with three canals. Kusaik et al. [91] reported the root morphology of mandibular premolars in female Polish patients with Turners syndrome, based on orthopantomogram X-ray images. They reported two-rooted mandibular teeth in 31–34% first premolars and 31–39% second premolars, which is much higher than that reported in general populations (<5%).

## 4. Discussion

Several factors contribute to variations found in the root and root canals that include ethnicity and gender. Scott and Turner [92], in their anthropological review, described the accessory root of first mandibular premolars as Tome's root, which showed a high incidence of greater than 25% in ethnic Australians and sub-Sahara African populations. Contrastingly, American Arctic, New Guinea, Jomon, and Western Eurasian populations had a lower incidence of the accessory root (0 to 10%). Such anthropological data could provide valuable information regarding the likelihood of an additional root in mandibular premolars of specific ethnic populations. Thus, the role of genetics should also not be underestimated in determining anatomic variations of human teeth.

Numerous methods have been used for studying root canal anatomy, including replication techniques [93], ground sections [94], clearing techniques [95], and radiography [96]. Advanced modes of radiographic imaging and analysis have allowed for in-depth knowledge of pulp space anatomy in three dimensions and allowed for identification of rare aberrations. These methods include spiral computed tomography (SCT) [16, 21], micro-computed tomography (micro CT) [24, 28, 97], and cone beam computed tomography (CBCT) [25–27]. Study design differences and the various origins of the investigated teeth could account for the highly variable results.

*Ex vivo* anatomic investigations, although the most widely used, inherently have certain shortcomings. It involves evaluation of extracted teeth that are frequently difficult to collect in sufficient numbers along with known specifics like age, gender, and so forth. Furthermore, most extracted teeth collected are severely damaged leading to difficulties in determining accurately the tooth notation. An additional negative impact if only sound teeth are selected is selection bias [25, 30]. Additionally, not all anatomic studies assessing mandibular premolars have reported the root and root canal configuration. Either one of these has been described with most studies discussing the root canal configuration (Tables 1 and 2). It would be of much more analytic and scientific value if the maximum data could be presented from the analyzed teeth, especially when the methodology leads to destruction of the samples. Thus, although a large number of mandibular premolars have been analyzed in anatomic studies, maximum extrapolation is lacking. CBCT imaging by means of its minimal radiation exposure, sample preservation, accuracy, and three-dimensional data acquisition of multiple teeth, could overcome the shortfalls of *ex vivo* methods and offer maximum information without solely depending on analysis of extracted teeth.

Differences in the method of analysis and data presentation could also contribute to an inaccurate perception of the incidence of variable anatomy. Data presented by number of patients instead of by number of teeth generally results in higher frequencies of the reported anomaly [14]. Also, the sample size studied in relation to the total sample of the population plays an important role in the overall ratio of variations. For instance, the only study analyzing the anatomy of a Kuwaiti population assessed only 20 mandibular first premolars of which 15% were two-rooted teeth and 40% contained two canals. Although these values are above the general weighted average or incidence among neighboring population groups, these findings may not reliably signify the accurate incidence of tooth anatomy within the population. Thus, studies of larger sample groups and streamlining data in the form of number of teeth could enhance comparison and also give a more dependable picture of the prevalent anatomy.

An interesting pattern that can be observed is that case reports of the second premolar by far outnumber those of the first premolar, especially in reported variations. This is contrary to the findings of epidemiologic studies, which reported an increased possibility of anatomic variation in mandibular first premolars. Despite these contrasting findings, anatomic studies serve as an indicator of the possible anatomic variations in analyzed population groups and should not be considered as the lone guiding principle. Wider anatomic variations are certainly possible in any tooth, as also in teeth that are less likely to show aberrations. On the other hand, case reports could be misleading to the clinician with regard to the incidence of the documented aberrations; however, their didactic value is of extreme importance. It allows the clinician to be in the best position to detect, discern, and diagnose various previously documented *in vivo* anatomic variations.

Pucci and Reig [98], in their monumental work Conductos Radiculares, concluded that the mandibular second premolar had two canals and two foramina 11.5% of the time, whereas the mandibular first premolar had branching canals, apical bifurcations, and trifurcations 26.5% of the time. Cleghorn et al. [99, 100], in a review of mandibular first and second premolars, presented findings regarding the number of roots, number of canals, and apical morphology. They reported that approximately 98% of mandibular first premolars were single-rooted with a single canal in 75.8% of the teeth. In cases of second premolars, almost all of the teeth in the anatomic studies were single-rooted (99.6%) with a single canal (91.0%). Comparatively, the findings of the present systematic review are more or less in agreement with these observations of Pucci and Reig [98] and Cleghorn et al. [99, 100].

The data analyzed in this systematic review is secondary data that is sourced from numerous previously published studies. Such secondary data is prone to drawbacks or biases that are inherent in the original data which could ultimately reflect in the results of this study as well. For instance, the method of analysis or the minimal number of teeth analyzed in a particular study. However, the intention was to provide the dental and endodontic fraternity, an interpretation of the vast data on mandibular premolars with a possibility of correlation to geographical origin and ethnicity that was not previously available.

The descriptions of the frequently occurring root and canal forms of permanent teeth are based largely on studies conducted in Europe and North America and relate to teeth of predominantly Caucasoid origin [1, 3]. The descriptions may not be wholly applicable to teeth of non-Caucasoid origin. The present systemic review provides additional and up-to-date information regarding the canal configuration of premolars and their apical exit patterns which allows for further comparison among different population groups around the globe. However, a very slight trend of more varied anatomy, that is, two or more roots or canals, could be seen in recent anatomic studies using modern imaging techniques, in as yet lesser analyzed population groups. Future documentation of root and canal anatomy in previously lesser studied geographical populations could show a trend of discrepancies between previously established weighted averages. However, it would be more appropriate and accurate to base successive comparisons of anatomic averages on ethnicities and demography's, as against the general norm of overall weighted averages.

## 5. Conclusions

1. The mandibular first premolar was more prone to bifurcation of canals (23–30%) terminating in multiple apical foramina (15–20%), as compared to second premolars.
2. The C-shaped canal pattern was most prevalent in first premolars of Chinese populations (upto 24%).
3. In second premolars, Caucasian, Indian, and Middle Eastern populations showed a higher prevalence of multiple canals (14–17%).
4. Type I canal configuration was most prevalent in both first (72.6%) and second premolars (83.65%).
5. A deep mesial radicular invagination was a common finding in multiple population groups in first premolars (13–27%).
6. Dens invaginatus was the most common developmental anomaly in first premolars.
7. There exists an association between ethnicity and root, root canal morphology across population groups in first and second premolars.

Recent imaging techniques and evaluation of wider populations have given a better insight regarding mandibular premolar anatomy and their inherent variations. Certain population and geographic groups have little or no data regarding mandibular premolar morphology, especially in South American, African, Australasian, and South East Asian populations. Although the studies that have been covered under this review did provide relevant information regarding root and root canal anatomy, other important data relating to apical anatomy, relationship of the anatomic apex with the apical foramen, precise location of bifurcations when present, mesiodistal width of roots, areas along the root that show narrowing or thinning, canal isthmuses, canal curvature, and so forth needs further evaluation and documentation in future research. Racial differences and its influence on pulp space anatomy should always be kept in mind.

## Supplementary Material

The data extrapolated through the statistics is attached as supplementary file.

## Figures and Tables

**Table 1:** Summary of the incidence of documented ethnic and population variations in the anatomy of mandibular first premolars.

Geographic and ethnic distribution	Materials and methods	No. of teeth in the study (*n*)	Root anatomy			Root canal anatomy				Root canal configuration (Vertucci)									Apical foramina			References
			1 root	2 roots	>2 roots	1 canal	2 canals	>2 canals	C-shaped	Type I	Type II	Type III	Type IV	Type V	Type VI	Type VII	Type VIII	Others types	1	2	3	
Caucasian																						
France	In vitro: radiograph and sectioning	341	90.6% (309)	6.4% (22)	3% (10)	68.9% (235)	31.1% (106)	0% (0)	0% (0)													Geider et al. [4], 1989
Germany	In vitro: examination	2369	99.3% (2352)	0.7% (17)	0% (0)																	Schulze. [5], 1970
Poland	In vitro: extraction and RCT	83				89.3% (74)	10.7% (9)	0% (0)	0% (0)													Rózyło et al. [6], 2008
USA	In vitro: clearing	400	100% (400)	0% (0)	0% (0)	70% (280)	25% (100)	5% (20)	0% (0)	70% (280)	0% (0)	4% (16)	1.5% (6)	24% (96)	0% (0)	0% (0)	0% (0)	0% (0)	74% (296)	26% (104)	0% (0)	Vertucci [7], 1894
	In vivo: radiographic examination	1002				81.8% (820)	18.2% (182)	0% (0)	0% (0)													Sabala et al. [8], 1994
	In vitro: grinding and examination under magnification	50				86% (43)	14% (7)	0% (0)	0% (0)										90% (45)	10% (5)	0% (0)	Green [9], 1973
	In vitro: sectioning	106				74% (78)	26% (28)	0% (0)	14% (15)	76% (80)	0% (0)	0% (0)	24% (26)	0% (0)	0% (0)	0% (0)	0% (0)	0% (0)	76% (80)	24% (26)	0% (0)	Baisden et al. [10], 1992
	In vitro: sectioning	32	100% (32)	0% (0)	0% (0)	35.5% (12)	62.5% (20)	0% (0)	0% (0)													Barrett. [11], 1925
	Extraction and radiograph	1287				66.9% (861)	32.7% (421)	0.4% (5)	0% (0)										80.7% (1039)	19.3% (248)	0% (0)	Zillich and Dowson [12], 1973
	In vitro: radiographs	156				95.5% (149)	4.5% (7)	0% (0)	0% (0)													Mueller et al. [13], 1933
	In vivo: radiographic examination	400	94.5% (378)	5.5% (22)	0% (0)	86.3% (345)	13.7% (55)	0% (0)	0% (0)													Trope et al. [14], 1986
Subgroup Ethnic Incidence		**6226**	**96.88%** **(3471)**	**2.52%** **(61)**	**0.6%** **(10)**	**75.42%** **(2586)**	**23.84%** **(935)**	**0.54%** **(25)**	**1.4%** **(15)**	**73%** **(360)**		**2%** **(16)**	**12.75** **(32)**	**12%** **(96)**					**80%** **(1490)**	**20%** **(383)**		

African																						
African American	In vivo: radiographic examination	400	83.8% (335)	16.2% (65)	0% (0)	67.2% (269)	32.8% (131)	0% (0)	0% (0)													Zillich and Dowson [12], 1973
Senegalese	In vivo: radiographic examination	412				81.3% (335)	15.1% (62)	3.6% (15)	0% (0)													Mbaye et al. [15], 2008
Subgroup Ethnic Incidence		**812**	**83.8%** **(335)**	**16.2%** **(65)**		**74%** **(604)**	**23.95%** **(193)**	**1.8%** **(15)**														
Indian																						
India	In vitro: SCT	100				80% (80)	11% (11)	0% (0)	2% (2)	80% (80)	9% (9)	3% (3)	2% (2)	4% (4)	0% (0)	0% (0)	0% (0)	2% (2)	94% (94)	6% (6)	0% (0)	Sandhya et al. [16], 2010
	In vivo: radiographic examination	1000	95.9% (959)	3.9% (39)	0.2% (2)					75.4% (754)	1% (10)	0% (0)	20.8% (208)	2.4% (24)	0% (0)	0% (0)	0.4% (4)	0% (0)	76.4% (764)	23.2% (232)	0.4% (4)	Iyer et al. [17], 2006
	In vitro: clearing	100				72% (72)	27% (27)	0% (0)	1% (1)	72% (72)	6% (6)	3% (3)	10% (10)	8% (8)	0% (0)	0% (0)	0% (0)	0% (0)	82% (82)	18% (18)	0% (0)	Velmurugan and Sandhya [18], 2009
	In vitro: clearing	40				50% (20)	50% (20)	0% (0)	0% (0)	50% (20)	5% (2)	5% (2)	25% (10)	12.5% (5)	2.5% (1)	0% (0)	0% (0)	0% (0)	82% (82)	18% (18)	0% (0)	Parekh et al. [19], 2011
	In vitro: clearing	138	97.1% (134)	2.89% (4)	0% (0)	67% (92)	33% (46)	0% (0)	0% (0)	67.4% (92)	8% (11)	3.7% (5)	3.9% (5)	17.4% (24)	0.7% (1)	0% (0)	0% (0)	0% (0)	77.8% (107)	22.2% (31)	0% (0)	Jain and Bahuguna [20], 2011
	In vitro: SCT	112	96.4% (108)	3.4% (4)	0% (0)	70.5% (79)	29.5% (33)	0% (0)	10.7% (12)	80% (90)	9% (10)	3% (3)	2% (2)	4% (4)	0% (0)	0% (0)	0% (0)	0% (0)	98% (110)	2% (2)	0% (0)	V. K. Sikri and P. Sikri [21], 1994
Subgroup Ethnic Incidence		**1490**	**96.47%** **(1201)**	**3.4%** **(47)**	**0.07%** **(2)**	**68%** **(343)**	**30%** **(137)**	**0%**	**3%** **(15)**	**71%** **(1108)**	**6%** **(48)**	**3%** **(16)**	**11%** **(237)**	**8%** **(69)**	**0.53%** **(2)**		**0.06%** **(4)**	**0.33** **(2)**	**85%** **(1239)**	**15%** **(307)**	**0.07%** **(4)**	

Mongoloid																						
China	In vitro: radiographs	100				64% (64)	34% (34)	2% (2)	0% (0)	78% (78)	6%` (6)	6% (6)	10% (10)	0% (0)	0% (0)	0% (0)	0% (0)	0% (0)	90% (90)	10% (10)	0% (0)	Walker [22], 1988
	In vitro: radiograph and sectioning	82				54% (44)	46% (38)	0% (0)	0% (0)													Lu et al. [23], 2006
	In vitro: Micro-CT	115				65.2% (75)	26.1% (40)	0% (0)	0% (0)	65.2% (75)	0% (0)	2.6% (3)	0% (0)	22.6% (26)	0% (0)	0.9% (1)	0% (0)	0% (0)	76.5% (88)	23.5% (27)	0% (0)	Liu et al. [24], 2013
	In vivo: CBCT	178	98% (174)	2% (4)	0% (0)	87.1% (155)	11.2% (20)	0.6% (1)	1.1% (2)	86.8% (151)	0% (0)	1.7% (3)	0% (0)	9.8% (17)	0% (0)	0% (0)	0.6% (1)	0% (0)	89.6% (160)	9.8% (17)	0.6% (1)	Tian et al. [25], 2012
	In vivo: CBCT	97				83.5% (81)	12.4% (12)	0% (0)	4.1% (4)	83.5% (81)	0% (0)	3.6% (3)	0% (0)	8.8% (8)	0% (0)	0% (0)	0% (0)	0% (0)	91.2% (88)	8.8% (9)	0% (0)	Liao et al. [26], 2011
	In vivo: CBCT	440	99.5% (439)	0.5% (1)	0% (0)	75.2% (340)	21% (92)	0.7% (3)	1.1% (5)	76% (335)	3.4% (15)	2.7% (12)	6.6% (21)	9.3% (41)	0% (0)	0% (0)	0.7% (3)	0% (0)	83.4% (367)	15.9% (70)	0.7% (3)	Tian et al. [27], 2013
	In vitro: Micro-CT	358							24% (86)													Fan et al. [28], 2008
Japan	In vitro: radiographs	516				86.2% (445)	13.8% (71)	0% (0)	0% (0)													Miyoshi et al. [29], 1977
	In vitro: Staining	139				80.6% (112)	15.1% (21)	4.3% (6)	0% (0)										80.6% (112)	19.4% (27)	0% (0)	Yoshioka et al. [30], 2004
Korea	In vivo: CBCT	797	99.9% (796)	0.1% (1)	0% (0)																	Park et al. [31], 2013
Subgroup Ethnic Incidence		**2822**	**99%** **(1409)**	**1%** **(6)**		**74.5%** **(1311)**	**22.5%** **(328)**	.**95%** **(12)**	**3.7%** **(97)**	**78%** **(720)**	**0.85%** **(21)**	**3%** **(27)**	**3.32%** **(31)**	**10.1%** **(92)**		**0.18%** **(1)**	**0.74%** **(4)**		**85%** **(905)**	**15%** **(160)**	**0.65%** **(4)**	

Middle East																						
Kuwait	In vivo: radiograph of RCT teeth	20	85.0% (17)	15% (3)	0% (0)	60% (12)	40% (8)	0% (0)	0% (0)										60% (12)	40% (8)	0% (0)	Zaatar et al. [32], 1997
Jordan	In vitro: clearing	500	97% (485)	3% (15)	0% (0)	58.2% (291)	39.8% (195)	2.6% (13)	0% (0)	58.2% (251)	4.8% (24)	1.4% (7)	14.4% (72)	16.8% (84)	0.8% (4)	1% (5)	0% (0)	2.6% (5)				Awawdeh and Al-Qudah [33], 2008
Iran	In vitro: sectioning	217	100% (217)	0% (0)	0% (0)	88.47% (192)	11.53% (25)	0% (0)	1.4% (3)	90% (195)	1.8% (4)	3.2% (7)	0.9% (2)	4.1% (9)	0% (0)	0% (0)	0% (0)	0% (0)	93.8% (203)	5% (11)	1.2% (3)	Khedmat et al. [34], 2010
	In vitro: clearing	163	98% (160)	2% (3)	0% (0)	70.6% (115)	27.8% (45)	1.2% (2)	2.4% (4)	70.6% (113)	1.9% (3)	3.8% (6)	3.8% (6)	16.9% (27)	1.2% (2)	0.6% (1)	0% (0)	1.2% (2)	77.5% (126)	22.5% (37)	0% (0)	Rahimi et al. [35], 2007
Turkey	In vitro: clearing	100	100% (100)	0% (0)	0% (0)	64% (64)	30% (30)	5% (6)	0% (0)										75% (75)	25% (25)	0% (0)	Çalişkan et al. [36], 1995
	In vitro: clearing	200				60.5% (121)	39.5% (69)	0% (0)	0% (0)	60.6% (121)	18.5% (37)	10.5% (21)	7% (14)	2.5% (5)	0% (0)	0% (0)	1% (2)	0% (0)	89.5% (179)	9.5% (19)	1% (2)	Sert and Bayirli [37], 2004
Subgroup Ethnic Incidence		**1200**	**96%** **(979)**	**6.67%** **(21)**		**67%** **(795)**	**31%** **(372)**	**1.46%** **(21)**	**0.63%** **(7)**	**69.85%** **(680)**	**6.75%** **(68)**	**4.73%** **(41)**	**6.53%** **(94)**	**9.85%** **(125)**	**0.5%** **(6)**	**0.4%** **(6)**	**0.25%** **(2)**	**0.95%** **(7)**	**79%** **(595)**	**20%** **(100)**	**0.44%** **(5)**	

Hispanic																						
Mexico	In vitro: radiograph	202				79.3% (140)	25.8% (62)	0% (0)	0% (0)										74.2% (150)	25.8 (52)	0% (0)	Pineda and Kuttler [38], 1972

Total no of teeth		12752	7395	200	12	6070	2027	73	181	2868	137	100	394	382	8	7	10	9	4349	1002	13	
Overall Incidence (%]			**97.21** **(%)**	**2.63** **(%)**	**0.16** **(%)**	**73.55** **(%)**	**23.55** **(%)**	**0.91** **(%)**	**2** **(%)**	**72.6** **(%)**	**3.73** **(%)**	**2.72** **(%)**	**10.72** **(%)**	**10.4** **(%)**	**0.22** **(%)**	**0.19** **(%)**	**0.27** **(%)**	**0.24** **(%)**	**81.08** **(%)**	**18.68** **(%)**	**0.24** **(%)**	

**Table 2:** Summary of the incidences of documented ethnic and population variations in the anatomy of mandibular second premolars.

Geographic and ethnic distribution	Materials and methods	No. of teeth in the study (*n*)	Root anatomy			Root canal anatomy				Root canal pattern (Vertucci)									Apical foramina			References
			1 root	2 roots	>2 roots	1 canal	2 canals	>2 canals	C-shaped	Type I	Type II	Type III	Type IV	Type V	Type VI	Type VII	Type VIII	Others types	1	2	3	
Caucasian																						
France	In vitro: radiograph and sectioning	328	97.6% (320)	2.4% (8)	0% (0)	86.6% (284)	13.4% (44)															Geider et al. [4], 1989
Germany	In vitro: analysis of extracted teeth	2089	99.85% (2086)	0.05% (1)	0.1% (2)																	Visser [41], 1948
Poland	In vitro: extraction and RCT	56				68.2% (38)	31.8% (18)	0% (0)	0% (0)													Rózyło et al. [6], 2008
U.S.A	In vitro: clearing	400	100% (400)	0% (0)	0% (0)	97.5% (390)	2.5% (10)	0% (0)	0% (0)	97.5% (390)	0% (0)	0% (0)	0% (0)	2.5% (10)	0% (0)	0% (0)	0% (0)	0% (0)	97.5% (390)	2.5% (10)	0% (0)	Vertucci [7], 1984
	In vitro: sectioning	32	100% (32)	0% (0)	0% (0)	65.6% (21)	34.4% (11)															Barrett [11], 1925
	In vitro: extraction and radiograph	906	96.6% (902)	0% (0)	3.4% (4)	87.5% (792)	12.5 (112)	0.4% (2)	0% (0)													Zillich and Dowson [12], 1973
	In vitro: grinding and examination under magnification	50				92% (46)	8% (4)	0% (0)	0% (0)										96% (48)	4% (2)	0% (0)	Green [9], 1973
Subgroup ethnic incidence		**3861**	**98.81%**	**0.49%**	**0.7%**	**82.9%**	**17.1%**	**0.07%**	**0%**	**97.5%**				**2.5%**					**96.75%**	**3.25%**		

African																						
Senegalese	In vivo: radiographic examination	408				86% (351)	12% (49)	2% (8)	0% (0)													Mbaye et al. [15], 2008

Indian																						
India	In vitro: clearing	40				80% (32)	20% (8)	0% (0)	0% (0)	80% (32)	0% (0)	0% (0)	2.5% (1)	17.5% (7)	0% (0)	0% (0)	0% (0)	0% (0)	80% (32)	20% (8)	0% (0)	Parekh et al. [19], 2011
	In vitro: SCT	96	97.9% (94)	2.1% (2)	0% (0)	87% (83)	13.5% (13)	0% (0)	0% (0)	80% (78)	9% (9)	3% (3)	2% (2)	4% (4)	0% (0)	0% (0)	0% (0)	0% (0)	94% (90)	6% (6)	0% (0)	V. K. Sikri and P. Sikri [21], 1994
Subgroup ethnic incidence		**136**	**97.9%**	**2.1%**	**0%**	**83.5%**	**16.75%**	**0%**	**0%**	**80%**	**4.5%**	**1.5%**	**2.25%**	**10.75%**					**87%**	**13%**		

Mongoloid																						
China	In vivo: CBCT	178	100% (178)	0% (0)	0% (0)	97.2% (173)	2.2% (4)	0% (0)	0.6% (1)	97.2% (173)	0% (0)	0% (0)	0.5% (1)	1.69% (3)	0% (0)	0% (0)	0% (0)	0.5% (1)				Tian et al. [25], 2012
Japan	In vitro: radiograph	40				97.9% (39)	2.1% (1)	0% (0)	0% (0)													Miyoshi et al. [29], 1977
Korea	CBCT	789	99.4% (784)	0.6% (5)	0% (0)																	Park et al. [31], 2013
Subgroup ethnic incidence		**1007**	**99.7%**	**0.3%**	**0%**	**97.5%**	**2.15%**	**0%**	**0.3%**	**97.2%**			**0.5%**	**1.69%**				**0.5%**				

Middle east																						
Kuwait	In vitro: radiograph of RCT teeth	64	95.6% (61)	4.4% (3)	0% (0)	95.3% (61)	4.7% (3)	0% (0)	0% (0)										95.3% (61)	4.7% (3)	0% (0)	Zaatar et al. [32], 1997
Jordan	In vitro: clearing	400	97.2% (389)	2.8% (11)	0% (0)	72% (288)	27.5% (110)	0.5% (2)	0% (0)	78% (288)	3.8% (15)	0.1% (4)	7.5% (30)	15.3% (61)	0% (0)	0% (0)	0% (0)	0.5% (2)	74.2% (337)	15.8% (63)	0% (0)	Awawdeh and Al-Qudah [33], 2008
Iran	In vitro: clearing	103	100% (103)	0% (0)	0% (0)	80.5% (83)	17.5% (18)	0% (0)	2% (2)	76.3% (76)	7.9% (9)	9.9% (11)	5.9% (7)	0% (0)	0% (0)	0% (0)	0% (0)	0% (0)	94.1% (97)	5.9% (6)	0% (0)	Rahimi et al. [35], 2007
	In vitro: clearing and sectioning	80				88.8% (71)	11.2% (9)	0% (0)	0% (0)	91.2% (73)	0% (0)	0% (0)	0% (0)	0% (0)	0% (0)	0% (0)	0% (0)	0% (0)	97.5% (78)	2.5% (2)	0% (0)	Hasheminia and Hashemi [39], 2005
	In vitro: clearing	137				94.2% (129)	5.8% (8)	0% (0)	0% (0)										75.9% (104)	24.1% (33)	0% (0)	Rahimi et al. [40], 2009
Turkey	In vitro: clearing	100	100% (100)	0% (0)	0% (0)	93.6% (94)	6.4% (6)	0% (0)	0% (0)	93.6% (94)	0% (0)	0% (0)	0% (0)	6.4% (6)	0% (0)	0% (0)	0% (0)	0% (0)	93.6% (94)	6.4% (6)	0% (0)	Çalişkan et al. [36], 1995
	In vitro: clearing	200	100% (200)	0% (0)	0% (0)	71% (142)	29% (58)	0% (0)	0% (0)	71% (142)	7% (14)	3.5% (7)	9% (18)	7% (14)	1.5% (3)	1% (2)	0% (0)	0% (0)	81.5% (163)	18.5% (37)	0% (0)	Sert and Bayirli [37], 2004
Subgroup ethnic incidence		**1084**	**98.56%**	**1.44%**	**0%**	**85.06%**	**14.59%**	**0.07%**	**0.29%**	**82.02%**	**3.74%**	**2.7%**	**4.48%**	**5.74%**	**0.3%**	**0.2%**		**0.1%**	**87.44%**	**11.13%**		

Hispanic																						
Mexico	In vitro: radiographs	250				98.8% (247)	1.2% (3)	0% (0)	0% (0)										98.8% (247)	1.2% (3)	0% (0)	Pineda and Kuttler [38], 1972
Total no of teeth		6746	5649	34	6	3364	489	12	3	1346	46	46	58	105	3	2	0	3	1741	179	0	
Overall incidence			99.28%	0.61	0.01%	86.9%	12.64%	0.31%	0.08%	83.65%	2.86%	2.86%	3.6%	6.52%	0.18%	0.12%	0%	0.18%	90.68%	9.32%	0%	

**Table 3:** Summary of exvivo studies reporting a deep mesial radicular invagination in mandibular premolars.

Population	Incidence	Tooth type	References
India	14%	1st premolar	Velmurugan and Sandhya [18], 2009
	14%	1st premolar	Sandhya et al. [16], 2010
	15.21%	1st premolar	Jain and Bahuguna [20], 2011

China	27.8%	1st premolar	Liu et al. [24], 2013

Jordan	17.6%	1st premolar	Awawdeh and Al-Qudah [33], 2008
	13.5%	2nd premolar	

Austria	15%	1st premolar	Robinson et al. [42], 2002

**Table 4:** Table enlisting documented case reports of mandibular first premolars with multiplicity of roots or root canals in teeth presenting.

Number of roots	Root canal anatomy	Diagnostic method	Country	Reference
1 root	2 canals	R/G	USA	England et al. [44], 1991
	2 canals	R/G	India	Shenoy et al. [45], 2013
	3 canals	R/G	Jamaica	Nallapati [46], 2005
	3 canals	R/G	Brazil	De Almeida-Gomes et al. [47], 2006

2 roots	2 canals	R/G	India	Kararia et al. [48], 2012
	3 canals	R/G	India	Poorni et al. [49] 2010
	3 canals	R/G	India	Moayedi and Lata [50], 2004
	N/A	R/G	USA	Milano et al. [51], 2002

3 roots	3 canals	R/G	India	Kakkar and Singh [52], 2012
	3 canals	R/G	China	Chan et al. [53], 1992
	3 canals	Extraction	USA	Fischer and Evans [54], 1992
	3 canals	Micro CT after extraction	Britain	Cleghorn et al. [55], 2008

N/A	3 canals	R/G	Germany	H$\ddot{\text{u}}$lsmann [56], 1990
N/A	3 canals	R/G	Australia	Yang [57], 1994

4 roots	4 canals	R/G	India	Vaghela and Sinha [58], 2013

—	4 canals	R/G	China	Du et al. [59], 2013

N/A: not available, R/G: radiograph, and micro-CT: microcomputed tomography.

**Table 5:** Table summarizing case reports of anatomical variations of roots and root canals in mandibular second premolars.

Number of roots	Root canal anatomy	Diagnostic mode	Country	Reference
1 root	3 canals	R/G	USA	Nallapati [46], 2005
	3 canals	R/G	India	N. Kararia and V. Kararia [60], 2013
	4 canals	R/G	Isreal	Holtzman [61], 1998
	4 canals	R/G	USA	Wong [62], 1991
	5 canals	R/G	Argentina	Macri and Zmener [63], 2000
	C-shaped	Micro-CT after extraction	Britain	Cleghorn et al. [55], 2008

2 roots	2 canals	R/G	India	Goswami et al. [64], 1997
	2 canals	R/G	India	Prakash et al. [65], 2008
	3 canals	R/G	China	Chan et al. [53], 1992
	3 canals	R/G	Germany	Rödig and Hülsmann [66], 2003
	3 canals	R/G	Belgium	De Moor and Calberson. [67], 2005
	3 canals	R/G	Iran	Lotfi et al. [68], 2008
	3 canals	R/G	Brazil	Soares et al. [69], 2009
	3 canals	R/G	Iran	Shokouhinejad [70], 2009
	3 canals	R/G	India	Aguiar et al. [71], 2010
	3 canals	R/G	India	Poorni et al. [49], 2010
	4 canals	R/G	USA	Bram and Fleisher [72], 1991
	4 canals	R/G	Saudi Arabia	Al-Fouzan [73], 2001
	4 canals	R/G	United Kingdom	Rhodes [74], 2001
	4 canals	R/G	Greece	Tzanetakis et al. [75], 2007
	3 canals	Extraction	USA	Fischer and Evans [54], 1992
	3 canals	R/G	Iran	Shalavi et al. [76], 2012
	3 canals	CBCT	Iran	Mokhtari et al. [77], 2013
	4 canals	Extraction	USA	Shapira and Delivanis [78], 1982

4 roots	4 canals	SCT	Indian	Sachdeva et al. [79], 2008
	4 canals	R/G	Greece	Farmakis [80], 2008

CBCT: cone beam computed tomography, Micro-CT: microcomputed tomography, SCT: spiral computed tomography, and R/G: radiograph.

**Table 6:** Summary of various developmental anomalies that have been reported in mandibular premolars.

Mandibular premolar	Developmental anomalies	Root/root canal anatomy	Diagnostic mode	Reference
1st premolar	Dens invaginatus	2 roots	R/G	Hartup [81], 1997
	Dens invaginatus	N/A	R/G	Bramante et al. [82], 1993
	Dens invaginatus	N/A	R/G	Tavano et al. [83], 1994
	Gemination	3 canals	R/G	Aryanpour et al. [84], 2002
	Dens invaginatus	1 root	R/G	Er et al. [85], 2007
	Dens invaginatus (bilateral)	N/A	R/G	Canger et al. [86], 2009

2nd premolar	Dens evaginatus	N/A	R/G	Koh et al. [87], 2001
	Fusion with mandibular first molar	5 canals	R/G	Tsesis et al. [88], 2003
	Fusion with supernumerary tooth	N/A	Clinical	Sathish Muthukumar et al. [89], 2012
	Taurodontism	3 canal	CBCT	Mokhtari et al. [77], 2013
	Taurodontism	5 canals	R/G	Demiryürek et al. [90], 2013

CBCT: cone beam computed tomography, R/G: radiograph, and N/A: not available.
